# A Novel D-Galacturonate Fermentation Pathway in *Lactobacillus suebicus* Links Initial Reactions of the Galacturonate-Isomerase Route With the Phosphoketolase Pathway

**DOI:** 10.3389/fmicb.2019.03027

**Published:** 2020-01-17

**Authors:** Laura C. Valk, Marijke A. H. Luttik, C. de Ram, Martin Pabst, Marcel van den Broek, Mark C. M. van Loosdrecht, Jack T. Pronk

**Affiliations:** Department of Biotechnology, Delft University of Technology, Delft, Netherlands

**Keywords:** galacturonic acid, pectin degradation, Entner–Doudoroff pathway, enrichment cultivation, heterolactic fermentation, anaerobic metabolism

## Abstract

D-galacturonate, a key constituent of pectin, is a ubiquitous monomer in plant biomass. Anaerobic, fermentative conversion of D-galacturonate is therefore relevant in natural environments as well as in microbial processes for microbial conversion of pectin-containing agricultural residues. In currently known microorganisms that anaerobically ferment D-galacturonate, its catabolism occurs via the galacturonate-isomerase pathway. Redox-cofactor balancing in this pathway strongly constrains the possible range of products generated from anaerobic D-galacturonate fermentation, resulting in acetate as the predominant organic fermentation product. To explore metabolic diversity of microbial D-galacturonate fermentation, anaerobic enrichment cultures were performed at pH 4. Anaerobic batch and chemostat cultures of a dominant *Lactobacillus suebicus* strain isolated from these enrichment cultures produced near-equimolar amounts of lactate and acetate from D-galacturonate. A combination of whole-genome sequence analysis, quantitative proteomics, enzyme activity assays in cell extracts, and *in vitro* product identification demonstrated that D-galacturonate metabolism in *L. suebicus* occurs via a novel pathway. In this pathway, mannonate generated by the initial reactions of the canonical isomerase pathway is converted to 6-phosphogluconate by two novel biochemical reactions, catalyzed by a mannonate kinase and a 6-phosphomannonate 2-epimerase. Further catabolism of 6-phosphogluconate then proceeds via known reactions of the phosphoketolase pathway. In contrast to the classical isomerase pathway for D-galacturonate catabolism, the novel pathway enables redox-cofactor-neutral conversion of D-galacturonate to ribulose-5-phosphate. While further research is required to identify the structural genes encoding the key enzymes for the novel pathway, its redox-cofactor coupling is highly interesting for metabolic engineering of microbial cell factories for conversion of pectin-containing feedstocks into added-value fermentation products such as ethanol or lactate. This study illustrates the potential of microbial enrichment cultivation to identify novel pathways for the conversion of environmentally and industrially relevant compounds.

## Introduction

Pectin, a commonly occurring polymer in plants, has a linear backbone of D-galacturonate residues or, in the case of rhamnogalacturonan, of alternating D-galacturonate and rhamnose residues. These backbones are decorated with sugars and carbonyl groups of D-galacturonate can be extensively esterified with methyl and acetyl-groups (Khanh et al., 1991; Searle-van Leeuwen et al., 1996). D-galacturonate accounts for approximately 70% of the mass of pectin (Mohnen, 2008). During microbial pectin degradation, it is released by the concerted action of pectinases and accessory enzymes (de Vries and Visser, 2001; Mohnen, 2008; Biz et al., 2014). Knowledge on the pathways involved in microbial fermentation of D-galacturonate is relevant for understanding degradation of plant biomass in anaerobic natural environments and in waste-water treatment processes. It is also highly relevant for the development of anaerobic microbial processes that can convert large-volume, pectin-rich agricultural residues such as apple pomace, citrus peel, and sugar-beet pulp into added-value products (Edwards and Doran-Peterson, 2012; Food and Agriculture Organization of the United Nations, 2016; Marzo et al., 2019).

Of three experimentally demonstrated pathways for D-galacturonate metabolism, two are found in prokaryotes and one in fungi. The prokaryotic oxidative pathway, first demonstrated in a *Pseudomonas* species (Zajic, 1959), converts D-galacturonate to α-ketoglutarate and CO_2_ via reactions that together reduce 2 moles of NAD(P)^+^ to NAD(P)H per mole of D-galacturonate (Zajic, 1959; Chang and Feingold, 1970). In contrast, the reaction sequence that converts D-galacturonate to pyruvate and glycerol in the fungal pathway requires the investment of 2 NAD(P)H per mole of D-galacturonate (Kuorelahti et al., 2005; Martens-Uzunova and Schaap, 2008; Zhang et al., 2011). Neither of these two routes enable redox-cofactor-neutral, fermentative pathways that generate ATP via substrate-level phosphorylation and they have hitherto only been encountered in microorganisms that are able to respire.

**FIGURE 1:**
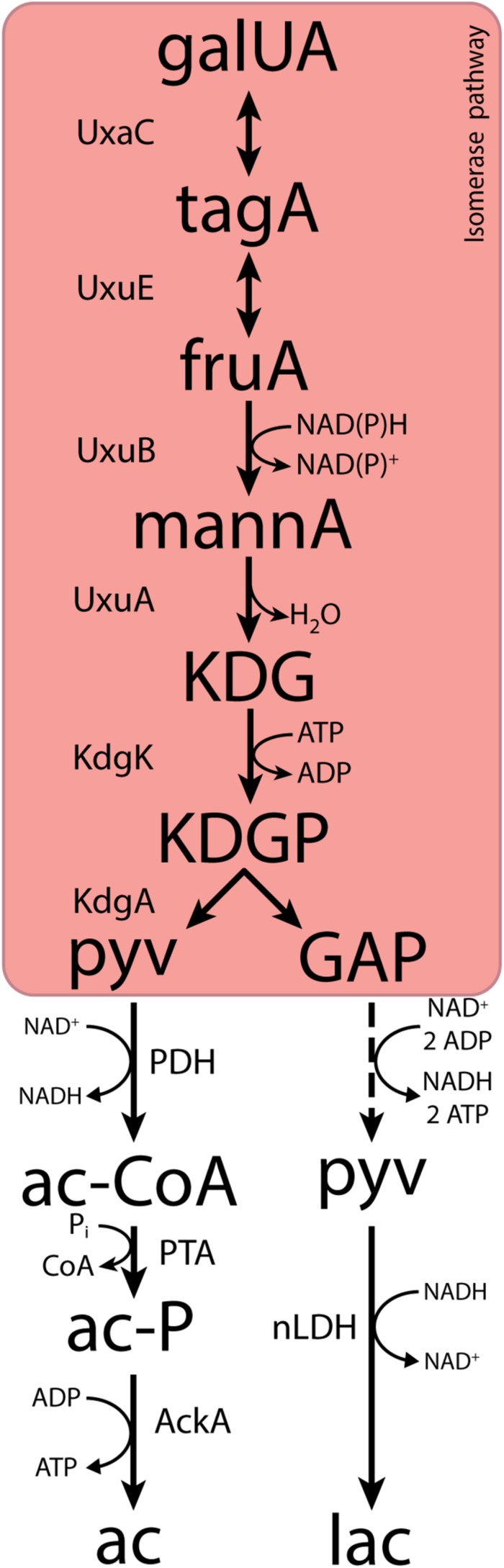
The canonical isomerase pathway for D-galacturonate fermentation. Dashed lines represent multiple conversions. Abbreviations indicate the following metabolites and enzyme activities: galUA, galacturonate; tagA, tagaturonate; fruA, fructuronate; mannA, mannonate; KDG, keto-deoxygluconate; KDGP, keto-deoxy-phosphogluconate; GAP, glyceraldehyde-3-phosphate; ac-CoA, acetyl-CoA; ac-P, acetyl-phosphate; pyv, pyruvate; lac, lactate; ac, acetate; UxaC, uronate isomerase; UxuE, tagaturonate 3-epimerase; UxuB, fructuronate reductase; UxuA, mannonate hydratase; KdgK, keto-deoxy-gluconate kinase; KdgA, keto-deoxy-phosphogluconate aldolase; PDH, pyruvate dehydrogenase; PTA, phosphotransacetylase; AckA, acetate kinase; nLDH, D-/L-lactate dehydrogenase.

Fermentative, anaerobic metabolism of D-galacturonate is firmly associated with a third pathway. First described in *Escherichia coli* (Kovachevich and Wood, 1955; Ashwell et al., 1960; Cynkin and Ashwell, 1960; Hickman and Ashwell, 1960; Smiley and Ashwell, 1960), this adapted Entner–Doudoroff or isomerase pathway converts D-galacturonate into pyruvate and glyceraldehyde-3-phosphate via 2-keto-3-deoxy-phosphogluconate (KDPG), the characteristic intermediate of the Entner–Doudoroff pathway for sugar dissimilation (Peekhaus and Conway, 1998). The canonical isomerase pathway (Figure 1) involves the activity via uronate isomerase (UxaC, EC 5.3.1.12), tagaturonate reductase (UxaB, EC 1.1.1.58), altronate dehydratase (UxaA, EC 4.2.1.7), and 2-keto-3-deoxy-gluconate kinase (KdgK, EC 2.7.1.45) and 2-keto-3-deoxy-phosphogluconate aldolase (KdgA, EC 4.1.2.14). Alternatively, conversion of tagaturonate into 2-keto-3-deoxy-gluconate can be catalyzed by tagaturonate 3-epimerase (UxuE, EC 5.1.2.7), fructuronate reductase (UxuB, EC 1.1.1.57), and mannonate dehydratase (UxuA, EC 4.2.1.8) (Kovachevich and Wood, 1955; Ashwell et al., 1960; Cynkin and Ashwell, 1960; Hickman and Ashwell, 1960; Smiley and Ashwell, 1960).

In both variants of the isomerase pathway, conversion of D-galacturonate into pyruvate and glyceraldehyde-3-phosphate requires the input of 1 ATP and 1 NAD(P)H. Further conversion of glyceraldehyde-3-phosphate via the lower part of the Embden–Meyerhof glycolysis yields one NADH and two ATP. Use of the isomerase pathway therefore enables redox-cofactor-neutral conversion of D-galacturonate into two moles of pyruvate, with a net ATP yield of 1 mol (mol galacturonate)^–1^ (Grohmann et al., 1994, 1998; Doran et al., 2000). This redox-cofactor neutrality constrains the range of fermentation products that can be generated from D-galacturonate. Acetate, which can be formed from pyruvate via redox-cofactor-neutral, ATP-yielding reactions, is typically found as the main product of microbial D-galacturonate fermentation (Grohmann et al., 1994; Doran et al., 2000; Valk et al., 2018; Kuivanen et al., 2019). For example, in a recent enrichment study on galacturonate performed at pH 8.0, the dominant organism “*Candidatus* Galacturonibacter soehngenii” predominantly produced acetate by a combination of galacturonate fermentation and acetogenesis (Valk et al., 2018). In bacteria engineered for ethanol production from D-galacturonate via the isomerase pathway, large amounts of more oxidized by-products are formed (Grohmann et al., 1994, 1998; Doran et al., 2000).

As yet undiscovered pathways for D-galacturonate fermentation, that allow for different fermentation product profiles, may exist in nature. Chemical decarboxylation of D-galacturonate to L-arabinose has been reported to occur under relatively mild conditions (Ruff, 1898; McKinnis, 1928; Link and Niemann, 1930) and the possibility that a similar enzyme-catalyzed reaction might occur has been proposed (Hirst, 1942; Gulland, 1943). Decarboxylation of L-arabinose could enable high-yield microbial production of compounds such as ethanol, lactate, or isobutanol. However, no experimental evidence for existence of the required D-galacturonate decarboxylase has yet been found in nature (Fan and Feingold, 1972; Marounek and Dušková, 1999).

Based on comparative genome analysis, Rodionova et al. (2012) suggested an alternative pathway for D-galacturonate metabolism involving epimerization of mannonate to gluconate. Subsequent conversion of gluconate by gluconokinase and 6-phosphogluconate dehydrogenase could also connect of D-galacturonate catabolism to pentose-phosphate metabolism. However, no experimental proof is available for *in vivo* activity of this pathway or for *in vitro* activity of the proposed mannonate epimerase.

Enrichment cultivation remains a powerful tool to select micro-organisms that are optimally adapted to individual and combined sets of environmental parameters from natural or industrial environments. Even when grown on a single substrate, population composition and product profiles in such cultures can be strongly influenced by process parameters such as culture pH, biomass retention time, temperature, and dynamic substrate-feeding strategies (Zoetemeyer et al., 1982a; Kleerebezem and van Loosdrecht, 2007; Johnson et al., 2009). It is well established that, in many fermentative microorganisms, cultivation at low pH induces a transition from the production of acids such as acetic or butyric acid to the formation of non-acidic products such as ethanol or butanol (Bahl et al., 1982; Grimmler et al., 2011). Similarly, enrichment cultivation at pH values below the pK_*a*_ of actic acid (4.76) has been found to select against acetic acid producing microorganisms, presumably due to an uncoupling of the pH gradient across the cytoplastic membrane by the undissociated acid (Eklund, 1983; Salmond et al., 1984).

This study explores whether anaerobic enrichment cultivation at pH 4.0 can lead to the discovery of novel pathways for D-galacturonate fermentation. To this end, anaerobic D-galacturonate-limited enrichment cultivation was performed in shake flasks, after which the dominant organism was isolated. Its product profile during anaerobic growth on D-galacturonate, which differed from that in previously described D-galacturonate fermenting bacteria, was characterized in anaerobic bioreactor batch and chemostat cultures and a combination of genomics, proteomics, and *in vitro* enzyme-activity analyses was used to investigate the responsible metabolic pathway.

## Results

### *Lactobacillus suebicus* Dominates Low-pH Anaerobic Enrichment Cultures on D-Galacturonate

Micro-organisms capable of fermenting D-galacturonate at low pH were enriched by serial transfer in anaerobic shake-flask cultures, grown at pH 4.0 on 4 g L^–1^ D-galacturonate. The initial cultures were inoculated with a mixture of rotting orange peels, orange-peel-enriched compost (Voragen et al., 2009). After two transfers to fresh medium, 16S-rRNA gene amplicon sequencing was performed. Based on these short 250-nucleotide amplicon sequences, the dominant taxon was the genus *Lactobacillus* (SINA database 1.2.11, Pruesse et al., 2012), with no other taxa represented by over 1% of the reads (Supplementary Figure S1). A pure culture was obtained by repeated anaerobic plating on galacturonate medium. The isolate grew anaerobically on D-galacturonate, L-arabinose, gluconate, and D-glucuronate in synthetic media supplemented with 0.4 g L^–1^ yeast extract (Supplementary Table S1).

Whole-genome long-read sequencing of the isolate, augmented with short-read sequencing, enabled assembly into one large (2.7 Mbp) and two smaller (89 and 28 kbp) contigs (Table 1). The full 16S-RNA gene sequence derived from the assembled genome sequence showed 100% identity with that of *Lactobacillus suebicus* (SINA database 1.2.11, Pruesse et al., 2012), a hetero-fermentative lactic acid bacterium previously isolated from apple and pear mashes (Kleynmans et al., 1989). Analysis with CheckM (Parks et al., 2015) yielded an estimated completeness of the genome sequence of 99.5%, with a GC content of 39.1% and 2811 predicted coding sequences (Table 1). These results are similar to previously published data by Nam et al. (2011) on the genome of *L. suebicus* DSM 5007 shown in Supplementary Table S2.

**TABLE 1:** Statistical data for the assembled and annotated genome sequence of *Lactobacillus suebicus* LCV1.

	***Lactobacillus suebicus* LCV1**
Genome size (Mbp)	2.8
Scaffolds	3
Contigs	3
Contigs N50	2673450
Max contig size	2673450
Completeness (%)	99.5
Contamination (%)	0
GC content (%)	39.1
Protein coding density (%)	85.5
Coding density sequences (CDS)	2811
rRNA copies	6

*Completeness and contamination were estimated with CheckM (Parks et al., 2015).*

### D-Galacturonate Fermentation by *L. suebicus* Yields Near-Equimolar Amounts of Acetate and Lactate

**FIGURE 2:**
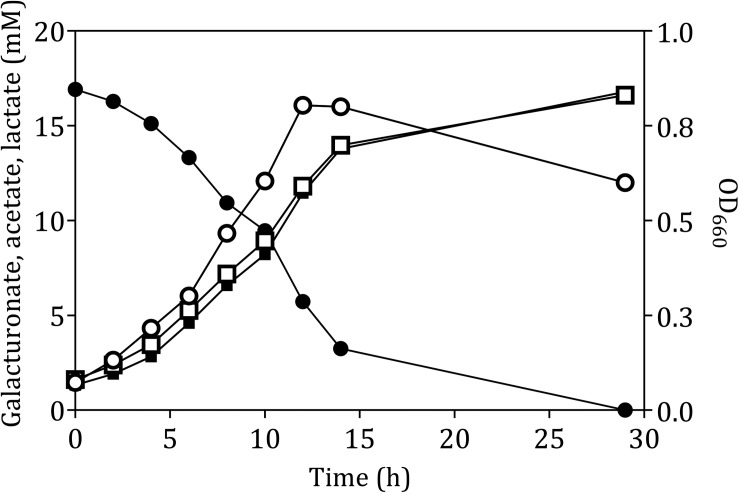
Anaerobic growth and product formation of in an anaerobic bioreactor batch culture of *L. suebicus* LCV1 on D-galacturonate (3.3 g L^–1^) at pH 4 and at 30°C. Symbols: ● D-galacturonate, ■ acetate, □ lactate, and ° optical density. The data shows one of two independent replicates; data from the second experiment are shown in Supplementary Figure S2.

The fermentation product profile of D-galacturonate-grown *L. suebicus* LCV1 was investigated in anaerobic bioreactor batch and chemostat cultures. In anaerobic bioreactor batch cultures, *L. suebicus* LCV1 exhibited a specific growth rate on D-galacturonate of 0.20 h^–1^ while acetate and lactate, the sole organic fermentation products, were formed at near-equimolar amounts (Figure 2). A similar product stoichiometry was observed in anaerobic, D-galacturonate-limited chemostat cultures grown at a dilution rate of 0.13 h^–1^, in which biomass-specific production rates of acetate and lactate were 6.0 ± 0.1 and 5.2 ± 0.1 mmol g_*x*_^–1^ h^–1^, respectively (Table 2). The biomass yield on D-galacturonate in these anaerobic chemostat cultures was 0.09 ± 0.0 g biomass (g galacturonate) ^–1^.

**TABLE 2:** Physiological parameters of anaerobic, galacturonate-grown chemostat cultures of *Lactobacillus suebicus* LCV1.

	**Biomass-specific conversion rates mmol (g biomass)** ^–^**^1^ h**^–^**^1^**	**Yield [mol_*i*_ mol (galacturonate)** ^–^**^1^]**
Galacturonate	−6.9 ± 0.0	
Acetate	6.0 ± 0.1	0.87 ± 0.01
Lactate	5.2 ± 0.1	0.75 ± 0.01
CO_2_	7.2 ± 0.1	1.04 ± 0.03
Biomass (g biomass g galacturonate^–1^)		0.09 ± 0.0

*Cultures were grown at pH 4, at a dilution rate of 0.13 h^–1^, and at 30°C. Biomass-specific rates of substrate consumption and product formation and product yields are presented as mean ± average deviations of measurements on two independent, steady-state chemostat cultures.*

### Enzymes Involved in the Lower Half of the Isomerase Pathway for D-Galacturonate Metabolism Are Absent From the Proteome of D-Galacturonate-Grown *L. suebicus* LCV1

In the canonical isomerase pathway for D-galacturonate fermentation, this substrate is generally first converted into two molecules of pyruvate via the isomerase pathway (Figure 1). Since this conversion is redox-cofactor neutral, it could only account for formation of equimolar amounts of acetate and lactate if pyruvate reduction to lactate by a lactate dehydrogenase (LDH) were stoichiometrically coupled to oxidative decarboxylation of pyruvate to acetate. The latter could, theoretically, be accomplished by the combined action of a pyruvate-dehydrogenase complex, phosphotransacetylase, and acetate kinase (Figure 1).

**FIGURE 3:**
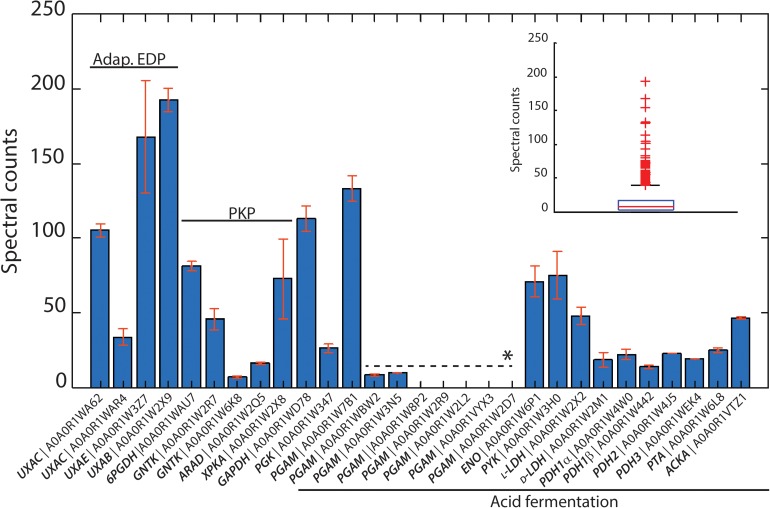
Identified proteins from the isomerase pathway (adapt. EDP), phosphoketolasse pathway (PKP) and acid fermentation plotted as normalized spectral counts in LC-MS-based proteomic analysis. The inset shows the box plot from all identified proteins within a range of 0–250 normalized counts. Only three protein hits were above this range, which are not shown. All proteins detected from the isomerase pathway, phosphoketolase pathway, and acid fermentation were above the 75th percentile of the complete proteome dataset. Bars represent the average value of two biological reactor duplicates, and error bars show the standard deviation between runs. ^∗^*Paralogous genes*.

The genome sequence of *L. suebicus* LCV1 showed a full complement of structural genes for the key enzymes of the isomerase pathway (Figure 3 and Table 3). Proteome analysis of D-galacturonate-grown cultures showed high levels of the enzymes of upper part of the adapted ED pathway [uronate isomerase (UxaC, EC 5.3.1.12), tagaturonate 3-epimerase (UxaE, EC 5.1.2.7) and fructuronate reductase (UxuB, EC 1.1.1.57)] (Table 3 and Figure 3). However, under the experimental conditions, mannonate dehydratase (UxuA, EC 4.2.1.8), 2-dehydro-3-deoxygluconokinase (KdgK, EC 2.7.1.45), and 2-dehydro-3-deoxyphosphogluconate aldolase (KdgA, EC 4.1.2.14), three key enzymes in the lower part of the isomerase pathway, were not detected in the proteome (Table 3).

**TABLE 3:** Annotated genes identified in the genome of *L. suebicus* LCV1, and associated expressed gene products identified by shot-gun proteomics for the two putative routes the isomerase pathway and the phosphoketolase pathway.

**Gene name**	**EC number**	**Gene**	**Identified protein sequence**
***Adapted Entner–Doudoroff pathway***			
Uronate isomerase^∗^	5.3.1.12	*uxaC*	A0A0R1WA62; A0A0R1WAR4
Tagaturonate 3-epimerase	5.1.2.7	*uxaE*	A0A0R1W3Z7
Fructonate reductase	1.1.1.57	*uxuB*	A0AR1W2X9
Mannonate dehydratase	4.2.1.8	*uxuA*	ND
2-Dehydro-3-deoxygluconokinase	2.7.1.45	*kdgK*	ND
2-Dehydro-3-deoxyphosphogluconate aldolase	4.1.2.14	*kdgA*	ND
***Phosphoketolase pathway***			
6-phosphogluconate dehydrogenase	1.1.1.44	*6pgd*	A0A0R1WAU7
Gluconate kinase^∗^	2.7.1.12	*gntK*	A0A0R1W2R7; A0A0R1W6K8
Ribulose-5-phosphate epimerase	5.1.3.4	*araD*	A0A0R1W2Q5
Xylulose-5-phosphate phosphoketolase	4.1.2.9	*xpkA*	A0AR1W2X8
***Acid fermentation***			
Glyceraldehyde-3-phosphate dehydrogenase	1.2.1.12	*gapDH*	A0A0R1WD78; A0A0R1W347
Phosphoglycerate kinase	2.7.2.3	*Pgk*	A0A0R1W7B1
Phosphoglycerate mutase^∗^	5.4.2.11	*Pgam*	A0A0R1WBW2;
			A0A0R1W3N5;
			A0A0R1W8P2;
			A0A0R1W2R9;
			A0A0R1W2L2;
			A0A0R1VYX3;
			A0A0R1W2D7
Enolase	4.2.1.11	*Eno*	A0A0R1W6P1
Pyruvate kinase	2.7.2.3	*Pyk*	A0A0R1W3H0
L-lactate dehydrogenase	1.1.1.27	*L-ldh*	A0AR1W2X2
D-lactate dehydrogenase	1.1.1.28	*D-ldh*	A0A0R1W2M1
Pyruvate dehydrogenase subunit E1α	1.2.4.1	*pdh E1*α	A0A0R1W4W0
Pyruvate dehydrogenase subunit E1β	1.2.4.1	*pdh E1*β	A0A0R1W442
Pyruvate dehydrogenase subunit E2	2.3.1.12	*pdh E2*	A0A0R1W4J5
Pyruvate dehydrogenase subunit E3	1.8.1.4	*pdh E3*	A0A0R1WEK4
Phosphate transferase	2.3.1.8	*Pta*	A0A0R1W6L8
Acetate kinase	2.7.2.1	*ackA*	A0A0R1VTZ1
Transhydrogenase	1.6.1.2	*pntAB*	A0A0R1W642;
			A0A0R1WE53;
			A0A0R1W6T3
NADH oxidase	1.6.3.4	*Nox*	A0A0R1WBZ1

*Gene name, EC number, genetic nomenclature, E-value based on SwissProt alignment with *Lactobacillus suebicus* DSM 5007 (BLASTP version 2.2.28 +, MicroScope platform v3.13.2) and their identified expressed protein products are listed below. ND = not detected. ^∗^Multiple annotated and expressed gene products detected.*

The three subunits of the pyruvate-dehydrogenase complex (PDH, EC 1.2.4.1; EC 2.3.1.12; EC 1.8.1.4), as well as phosphate transacetylase (PTA, EC 2.3.1.8), acetate kinase (AckA, EC 2.7.2.1), and both L- and D-LDH [L(+)-nLDH, EC 1.1.1.27; D-(-)-nLDH EC 1.1.1.28] were all detected in the proteome (Figure 3 and Table 3). These results indicated that, under the experimental conditions, enzymes for conversion of pyruvate to lactate and acetate were present in *L. suebicus* LCV1. However, absence of UxuA, KdgK, and KdgA in the proteome indicated that, in the anaerobic galacturonate-grown batch cultures, pyruvate formation from galacturonate did not occur via a complete, canonical isomerase pathway.

### Proteome Analysis and Activities of “Conventional” Enzymes in Cell Extracts Indicate Involvement of a Hybrid Isomerase/Phosphoketolase Pathway

The phosphoketolase (PK) pathway in lactic acid bacteria is a well-known route for hetero-fermentative dissimilation of sugars to equimolar amounts of lactate, ethanol, and CO_2_ (DeMoss et al., 1951; Gunsalus and Gibbs, 1952; Gibbs et al., 1955; Busse et al., 1961). It has previously been proposed that D-galacturonate metabolism via the initial reactions of the isomerase pathway can be linked to the PK pathway by epimerization of mannonate to gluconate (Rodionova et al., 2012), although this mechanism has not been experimentally demonstrated. Consistent with the involvement of such hybrid pathway, proteome analysis of galacturonate-grown cultures of *L. suebicus* LCV1 showed high levels of a gluconate kinase (GntK, EC 2.7.1.12), 6-phosphogluconate dehydrogenase (GndA, EC 1.1.1.44), and phosphoketolase (XpkA, EC 4.1.2.9), three key enzymes of the PK pathway (Table 3 and Figure 3).

*In vitro* activity assays were used to investigate the presence of known enzyme reactions involved in the proposed hybrid isomerase-PK pathway in galacturonate-grown *L. suebicus* LCV1. Under aerobic conditions, cell extracts rapidly oxidized NADH (Table 4). This activity was attributed to an NADH oxidase (NoxA, EC 1.6.3.4), for which a candidate gene (A0A0R1WBZ1, Table 3) and high expression levels of the encoded protein (Figure 3) were detected. Enzyme activity assays that involved NAD^+^ or NADH were therefore performed under anaerobic conditions. Fructuronate reductase (UxuB, EC 1.1.1.57), a key enzyme of the upper part of the isomerase pathway, was measured with D-tagaturonate and NADH or NADPH as substrates and showed an activity of 0.99 ± 0.01 and 0.2 ± 0.01 μmol (mg protein)^–1^ min^–1^, respectively. Cell extracts also showed high activities of NADP^+^-dependent 6-phosphogluconate dehydrogenase (GndA, EC 1.1.1.44; Table 4), the first enzyme of the PK pathway. Activities of NAD^+^-dependent LDH [L(+)-nLDH, EC 1.1.1.27; D-(-)-nLDH EC 1.1.1.28] in cell extracts of D-galacturonate-grown *L. suebicus* LCV1 (Table 4) were similar to those observed in cell extracts of sugar-grown cultures of other Lactobacilli (de Vries et al., 1970; Thomas et al., 1979). Pyruvate-dehydrogenase activity in cell extracts was below detection limit (Table 4).

**TABLE 4:** Specific enzyme activities of key enzymes of the isomerase and phosphoketolase pathways of cell extract of galacturonate-grown *L. suebicus* LCV1 (*D* = 0.13 h^–1^, *T* = 30°C, and pH 4).

**Enzyme name**	**Enzyme**	**Co-factor**	**Substrate**	**Specific activity (μmol mg_*protein*_**^–^**^1^ min**^–^**^1^)**
Fructuronate reductase	UxuB	NADPH	Tagaturonate	0.24 ± 0.1
Fructuronate reductase	UxuB	NADH	Tagaturonate	0.99 ± 0.1
Gluconate kinase	GntK	ATP and NADP^+^	Mannonate	0.08 ± 0.0
6-phospholgluconate dehydrogenase	6PGD	NADP^+^	6-phospho-gluconate	1.18 ± 0.1
Lactate dehydrogenase	nLDH	NADH	Pyruvate	5.80 ± 0.7
NADH oxidase	NOX	NADH	Oxygen	0.19 ± 0.0
Pyruvate dehydrogenase	PDH	NAD	Pyruvate	<0.05

*Both gluconate kinase (GntK) and fructuronate reductase (UxuB) were measured by a coupled reaction with a naturally expressed epimerase [6-phosphomannonate 2-epimerase and tagaturonate 3-epimerase (EC 5.1.2.7, UxuE), respectively]. Mean ± standard deviations were derived from duplicate experiments.*

### Demonstration of Mannonate Kinase and 6-Phosphomannonate-2-Epimerase Activities in Cell Extracts

**FIGURE 4:**
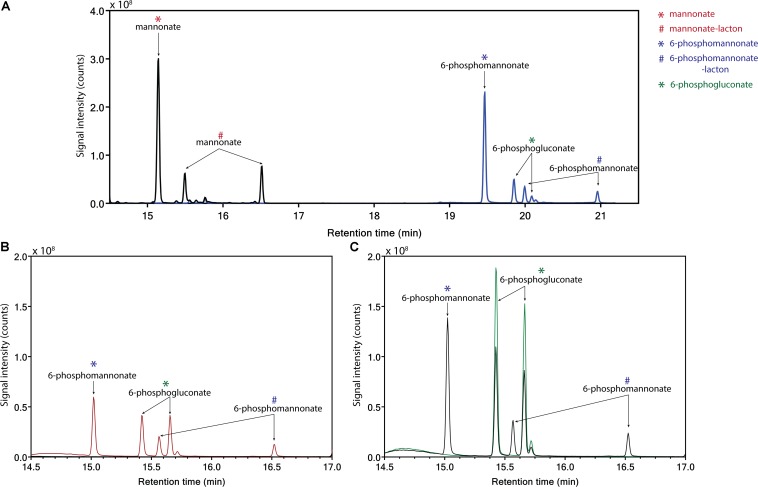
Identification of products from the conversion of mannonate or 6-phosphogluconate, in the presence and absence of ATP, with cell extract from *L. suebicus* LCV1, grown on galacturonate in anaerobic chemostat cultures (*D* = 0.13 h**^–^**^1^, 30°C, pH 4). **(A)** 0.5 h incubation of mannonate with cell extract in the absence (black line) and presence of ATP (blue line), **(B)** incubation of mannonate with cell extract and ATP, start of incubation (black line), and sample taken after 3 h of incubation (red line), and **(C)** incubation of 6-phosphogluconate, start of incubation (green line), and sample taken after 3 h of incubation (black line). For each experiment, one of two of independent biological duplicate experiments is shown. Data for the duplicate experiments are shown in Supplementary Figure S3. Different retention times of compounds in the panels are caused by a reduced column length for the experiments shown in **C**. Retention times of the standards (mannonate and 6-phosphogluconate) and the annotated GC-MS profile of mannonate are shown in Supplementary Figures S4, S5, respectively. ^∗^sugar acid; ^#^lacton-variant of the sugar acid.

Conversion of mannonate to 6-phosphogluconate, a key conversion in the proposed hybrid isomerase-PK pathway, has not previously been demonstrated. To investigate whether this conversion occurs in *L. suebicus* LCV1, cell extracts were incubated with different substrates and products were analyzed by GC-MS. Incubation (0.5 h) of cell extracts with mannonate did not lead to formation of gluconate (Figure 4A, black line). When ATP was also added, mannonate was predominantly converted to 6-phosphomannonate, thus indicating the activity of a mannonate kinase in the cell extracts. In addition, small amounts of 6-phosphogluconate were formed (Figure 4A, blue line). Upon prolonged incubation of cells extract with mannonate and ATP, formation of 6-phosphomannonate was clearly observed (Figure 4B, red line). In addition, incubation of cell extracts with 6-phosphogluconate led to the formation of 6-phosphomannonate (Figure 4C, black line). These results demonstrated the presence, in galacturonate-grown *L. suebicus*, of 6-phosphomannonate 2-epimerase activity (for reaction, see Supplementary Figure S6).

**FIGURE 5:**
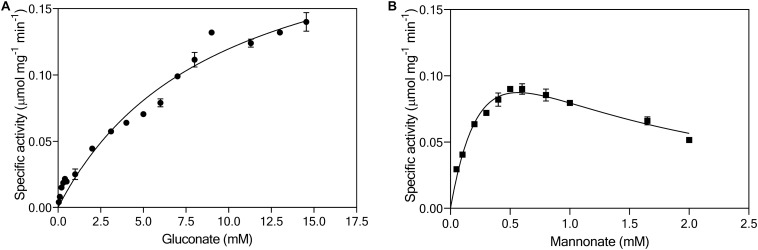
Effect of **(A)** gluconate (●, mM) and **(B)** mannonate concentrations (■, mM) on the specific enzymatic activity (μmol mg_*protein*_**^–^**^1^ min**^–^**^1^) of gluconate kinase and mannonate kinase, respectively, in cell extracts of *L. suebicus* LCV1, pre-grown on galacturonate in anaerobic chemostat cultures (*D* = 0.13 h**^–^**^1^, 30°C, pH 4). The average ± mean deviations were derived from duplicate measurements.

Continuous enzyme-activity assays with cell extracts, in which activity of gluconate kinase was coupled to 6-phosphogluconate dehydrogenase, revealed a K_*M*_ for gluconate of 10 mM and a *V*_*max*_ of 0.24 μmol (mg protein)^–1^ min^–1^ (Figure 5A). This K_*M*_ was two to three orders of magnitude higher than reported for bacterial gluconate kinases (Zachariou and Scopes, 1985; Izu et al., 1996; Tong et al., 1996). The same continuous assay, using the native, unidentified 6-phosphomannonate epimerase activity as a coupling enzyme, was used to investigate the kinetics of mannonate kinase in *L. suebicus* LCV1. Although this assay did not contain the demonstrated excess of coupling-enzyme activity required for reliable kinetic analyses of mannonate kinase activity, it indicated the presence of a high-affinity mannonate kinase activity (Figure 5B, estimated K_*M*_ of 0.4 mM). The observed maximum reaction rate and apparent substrate inhibition at higher substrate concentrations could reflect properties of either the mannonate kinase or of the unidentified epimerase enzyme(s) under the conditions of the *in vitro* assay.

## Discussion

Enrichment cultivation at low pH enabled the isolation of a lactic acid bacterium with a product profile that had not previously been observed during anaerobic fermentation of D-galacturonate. In contrast to previously described D-galacturonate-fermenting organisms, the isolate, identified as a strain of *L. suebicus*, did not produce acetate as single major fermentation product but, instead, produced lactate and acetate at near-equimolar ratios. Due to its lower pK_*a*_ and lower lipid solubility, the uncoupling effect of lactate on the proton gradient across biological membranes is much less pronounced than that of acetic acid (Leyer et al., 1995; Abbott et al., 2008). Our results are therefore in line with previous studies in which enrichment cultivation at low pH values generated a negative selective pressure against the formation of acetate as predominant fermentation product (Zoetemeyer et al., 1982b; Fang and Liu, 2002; Horiuchi et al., 2002; Temudo et al., 2007). At low extracellular concentrations of lactate, some anaerobic bacteria can couple lactate export via a proton symporter to energy conservation (Ten Brink and Konings, 1986; Trchounian and Trchounian, 2019). If active in *L. suebicus*, such an “end-product-efflux” mechanism might provide an additional, condition-dependent advantage over formation of only acetate.

Lactobacilli are known for their ability to ferment a wide range of sugars under mildly acidic conditions (van Hylckama Vlieg et al., 2006; Siezen et al., 2011). However, metabolic pathways for D-galacturonate metabolism and the resulting product profiles in Lactobacilli have not previously been studied in detail. In the context of the present study, it is relevant to note that apple and pear mashes, from which *L. suebicus* has previously been isolated, are rich in pectin and that, during growth on sugars, the metabolism of *L. suebicus* was characterized as heterofermentative (Kleynmans et al., 1989).

We initially hypothesized that the observed product stoichiometry reflected a simultaneous, redox-cofactor-balanced reduction and oxidative decarboxylation of pyruvate, generated via a canonical isomerase pathway for D-galacturonate metabolism (Figure 1; Wagner et al., 2005; Valk et al., 2018). Although pyruvate dehydrogenase (PDH) complexes are typically inhibited at high NADH/NAD^+^ ratios (Snoep et al., 1992, 1993), such a concerted action of the PDH complex and an NAD^+^-linked LDH has been implicated in fermentation of pyruvate by *Oenococcus oeni* and *Leucococcus mesenteroides* (Hansen and Henning, 1966; Wagner et al., 2005). However, its involvement inD-galacturonate fermentation by *L. suebicus* was rejected based on the absence of key enzymes of the lower half of the canonical isomerase pathway in the proteome of galacturonate-grown cultures (Table 3 and Figure 3).

**FIGURE 6:**
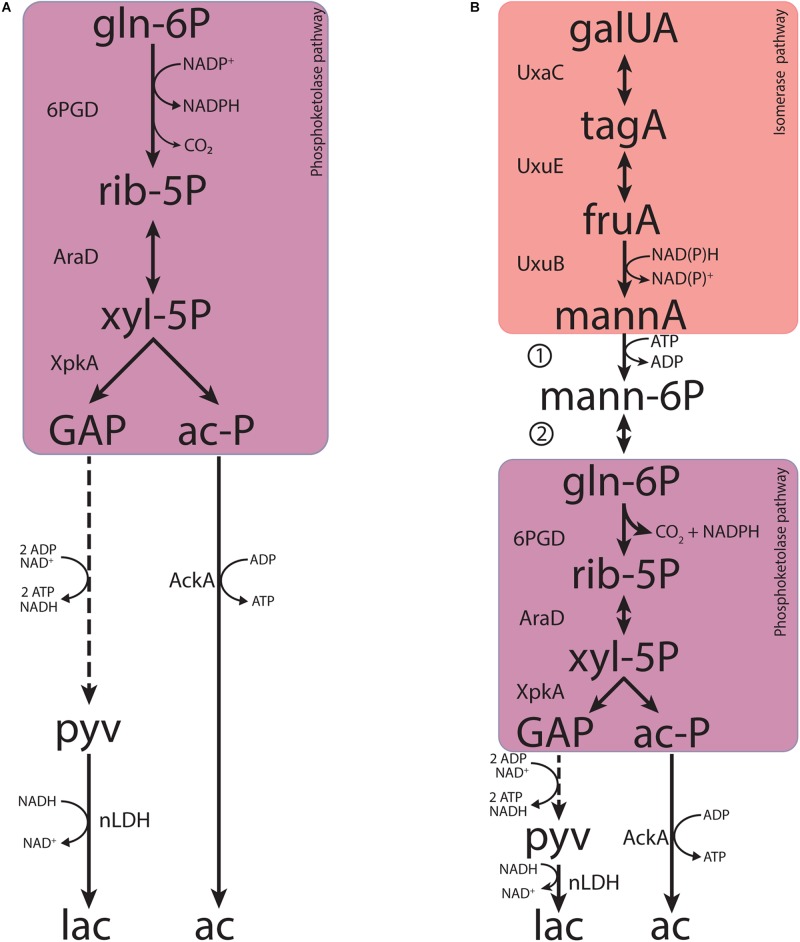
Metabolic pathways contributing to fermentative galacturonate fermentation in *L. suebicus* LCV1 as discussed in this paper. **(A)** Phosphoketolase pathway and **(B)** novel pathway proposed in this study that combines known reactions of the upper part of the canonical isomerase pathway and the phosphoketolase pathway, coupled by the concerted action of a mannonate kinase and a 6-phosphomannonate 2-epimerase. Dashed lines represent multiple conversions. Abbreviations indicate the following metabolites and enzyme activities: galUA, galacturonate; tagA, tagaturonate; fruA, fructuronate; mannA, mannonate; mann-6P, 6-phosphomannonate; gln-6P, 6-phosphogluconate; rib-5P, ribulose-5-phosphate; xyl-5P, xylulose-5-phosphate; GAP, glyceraldehyde-3-phosphate; ac-P, acetyl-phosphate; pyv, pyruvate; lac, lactate; ac, acetate; UxaC, uronate isomerase; UxuE, tagaturonate 3-epimerase; UxuB, fructuronate reductase, 1, mannonate kinase; 2, 6-phosphomannonate 2-epimerase; 6PGD, 6-phosphogluconate dehydrogenase (decarboxylating); AraD, ribulose-5-phosphate 4-epimerase; XpkA, phosphoketolase; AckA, acetate kinase; nLDH, D-/L-lactate dehydrogenase.

Based on investigations on *Thermotoga maritima*, a deep-branching, anaerobic, hyperthermophilic bacterium able to degrade a wide range of carbohydrates including pectin (Nelson et al., 1999), Rodionova et al. (2012) proposed that the initial reactions of the isomerase pathway for galacturonate might be coupled to the PK pathway (Figure 6A) by gluconate 2-epimerase (*gntE*), and a gluconate kinase (*gntK*, EC 2.1.7.12). This proposal was based on the organization of hexuronate and pectin utilization loci, in which *gntE*, which was assumed to encode the epimerase activity, was found between the fructuronate reductase (*uxuA*, EC 1.1.1.58) and gluconate kinase genes. A homology search indicated that *gntE* homologs also occurred in other bacterial genera, including *Lactobacillus*, *Acidobacterium*, and *Terriglobus* (Rodionova et al., 2012). However, no experimental evidence was provided to show that *gntE* indeed encoded the proposed epimerase.

Discontinuous and continuous enzyme activity assays and product identification by GC-MS showed that, instead of a direct epimerization of mannonate to gluconate followed by phosphorylation of gluconate (Rodionova et al., 2012), *L. suebicus* LCV1 first phosphorylated mannonate to 6-phosphomannonate, followed by an epimerization to 6-phosphogluconate (Figure 6B). This newly discovered conversion, which couples the isomerase pathway to the PK pathway, involves two enzyme activities, a mannonate kinase and a 6-phosphomannonate/6-phoshogluconate 2-epimerase, that have not previously been described.

The BRENDA database (Schomburg et al., 2017; Jeske et al., 2019) does not list specific mannonate kinases, nor does it list mannonate as a known substrate for gluconate kinases. Indeed, *E. coli* gluconate kinase (GntK, EC 2.7.1.12) was reported to be unable to use D-mannonate as substrate (Cohen, 1950). The high K_*M*_ of gluconate kinase activity and the low apparent K_*M*_ of mannonate kinase in cell extracts (Figure 4) strongly suggest that at least one of the two *L. suebicus* genes with strong homology to gluconate kinase genes (A0A0R1W2R7, A0A0R1WAU7), whose products were both detected in the proteome of galacturonate-grown cells (Table 3 and Figure 3), encodes a kinase with a much higher affinity for mannonate than for gluconate.

Four Structural Classification of Proteins (SCOP) families harbor 2-epimerases; NAD^+^-dependent epimerases or dehydratases (CEP1), *N*-acylglucosamine epimerases (CEP4), NanE-like (CEP5), and UDP-*N*-acetylglucosamine 2-epimerases (CEP9). All these epimerases act on compounds with a CDP or UDP side-group or on dimers (Senoura et al., 2009; Van Overtveldt et al., 2015). No homology to structural genes for either of the enzyme groups was identified in the *L. suebicus* LCV1 genome. Based on co-localization with hexuronate and D-gluconate fermentation pathway genes, Rodionova et al. (2012) proposed that the TM0042 gene in *T. maritima* encoded a gluconate-mannonate 2-epimerase. The predicted protein sequence showed similarity to an aldose 1-epimerase (EC 5.1.3.3) or a 6-phosphoglucose 1-epimerase (EC 5.1.3.15), but no biochemical evidence for its activity was provided. A homologous gene was identified in *L. suebicus* LCV1 (A0A0R1W1H9) and although the gene was not co-localized with genes of the D-galacturonate fermentation pathway, the encoded protein product was successfully identified with high sequence coverage in the D-galacturonate-grown cultures. Further research is required to investigate whether this protein encodes the 6-phosphomannonate 2-epimerase active in these cultures.

Consistent with the literature on other organisms (Cohen, 1950; McNair Scott and Cohen, 1952, 1956), activity of 6-phosphogluconate dehydrogenases (GndA, EC 1.1.1.44) in cell extracts of *L. suebicus* showed high activities NADP^+^ as electron acceptor. While fructuronate reductases typically use NADH as co-factor (Hickman and Ashwell, 1960; Portalier and Stoeber, 1972; Mandrand-Berthelot and Lagarde, 1977; Linster and van Schaftingen, 2004), cell extracts of *L. suebicus* LCV1 also showed activity of this enzyme with NADPH as electron donor (Table 3). A matching cofactor use off these two enzymes, possibly further facilitated by the expression of a transhydrogenase (PntAB; EC 1.6.1.2, A0A0R1W642; A0A0R1WE53; A0A0R1W6T3), enables redox-cofactor balancing in the integrated isomerase-PK pathway (Figure 6B).

Enzyme purification and characterization, possibly combined with heterologous expression studies and/or generation and analysis of *L. suebicus* mutants, should resolve the genetic basis for the mannonate kinase and 6-phosphomannonate/6-phosphogluconate-2-epimerases in *L. suebicus* LCV1. In addition to generating fundamental knowledge on anaerobic metabolism in natural environments of galacturonate, a ubiquitous monomer in plant biomass, such studies are likely to have a considerable industrial relevance. In contrast to the canonical isomerase pathway (van Maris et al., 2006), the integrated isomerase-PK pathway demonstrated in this study enables redox-cofactor neutral conversion of D-galacturonate to a ribulose-5-phosphate and CO_2_ (Figure 6B). Linking these reactions to the non-oxidative pentose-phosphate pathway would pave the way for new metabolic engineering strategies for high-yield, anaerobic conversion of galacturonate-containing feedstocks to compounds such as ethanol, isobutanol, or lactate.

## Materials and Methods

### Growth Media

Unless stated otherwise, liquid medium contained (g L^–1^): D-galacturonate 4.3; NH_4_Cl 1.34; KH_2_PO_4_ 0.78; Na_2_SO_4_.10H_2_O 0.130; MgCl_2_.6H_2_O 0.120; FeSO_4_. 7H_2_O 0.0031; CaCl_2_ 0.0006; H_3_BO_4_ 0.0001; Na_2_MoO_4_. 2H_2_O 0.0001; ZnSO_4_.7H2O 0.0032; CoCl_2_.H_2_O 0.0006; CuCl_2_.2H_2_O 0.0022; MnCl_2_.4H_2_O 0.0025; NiCl_2_.6H_2_O 0.0005; EDTA 0.10, tryptone (BD Bacto Difco, ThermoFisher Scientific, United States) 0.6, yeast extract (BD Bacto Difco, ThermoFisher Scientific, United States) 0.4, and vitamin solution (Verduyn et al., 1992) 1 mL L^–1^; 19 L of mineral solution with yeast extract (concentration adjusted to the final volume, 20 L) was autoclaved for 20 min at 121°C. Subsequently, 86 g D-galacturonate, 8 g tryptone, and 20 mL vitamin solution in 1 L demineralized water was filter sterilized (0.2 μm Mediakap Plus, Spectrum Laboratories, Rancho Dominguez, United States) and added to the autoclaved medium. For bioreactor cultivation, 0.075 mL L^–1^ Pluronic PE 6100 antifoam (BASF, Ludwigshafen, Germany) was added to the mineral medium before autoclaving.

### Isolation and Maintenance

Anaerobic 50-mL shake flasks containing 30 mL liquid medium were inoculated with 2% (v/v) of a mixed inoculum consisting of rumen content of a grass-fed cow, provided by an artisanal butcher (Slager Jonkers, Est, Netherlands), rotting orange peels and orange-peel-enriched compost. Triplicate shake flask cultures were grown at 30°C in an anaerobic chamber (Bactron III, Shell Lab, Cornelius, NC, United States, gas composition 89% N_2_, 6% CO_2_, and 5% H_2_) and transferred once, upon depletion of the carbon source. After the second enrichment phase, the enrichment cultures were plated on 1% agar plates (4 g L^–1^ D-galacturonate, 0.4 g L^–1^ yeast extract, pH 5) under anaerobic conditions. Single colonies were re-streaked thrice and grown in shake flasks on liquid medium. Stocks were stored in 30% (v/v) glycerol at −80°C.

### Bioreactor Cultivation

Batch and chemostat cultures were grown in 1.2 L laboratory bioreactors (Applikon, Delft, Netherlands) at a temperature of 30°C. Reactors were stirred at 300 r/min and, to maintain anaerobiosis, sparged with nitrogen gas at a flow rate of 120 mL min^–1^. Culture pH was controlled at pH 4.0 ± 0.1 by automatic titration (ADI 1030 Biocontroller, Applikon, Delft, Netherlands) with 1 M NaOH. For chemostat cultivation, the dilution rate was set at 0.13 ± 0.01 h^–1^ and a working volume of 0.50 L was maintained by a peristaltic effluent pump (Masterflex, Cole-Parmer, Vernon Hills, IL, United States) coupled to a level sensor.

### Metabolite Analysis in Culture Supernatants

Cell-free supernatants were obtained by centrifugation (Heraeus Pico Microfuge, ThermoFisher Scientific, Waltham, NC, United States) of culture samples. Concentrations of galacturonate and extracellular metabolites were analyzed on an Agilent 1100 Affinity HPLC (Agilent Technologies, Santa Clara, CA, United States) equipped with an Aminex HPX-87H ion-exchange column (BioRad, Hercules, CA, United States), operated at 60°C with a mobile phase of 5 mM H_2_SO_4_ and at a flow rate of 0.6 mL min^–1^. Concentrations of CO_2_ and O_2_ in bioreactor exhaust gas were measured with a Prima BT Bench Top mass spectrometer MS (Thermo Scientific Fisher, Waltham, NC, United States) after cooling the gas with a condenser (4°C).

### Biomass Dry Weight

20 mL of culture broth samples was filtered over pre-dried and pre-weighed membrane filters (0.2 μm Supor-200, Pall Corporation, New York, NY, United States), washed with demineralized water, dried in a microwave oven (Robert Bosch GmbH, Gerlingen, Germany) for 20 min at 360 W, and reweighed. Carbon and electron balances were constructed based on the number of carbon atoms and electrons per mole, with an assumed biomass composition of CH_1_._8_O_0_._5_N_0_._2_ (Roels, 1983).

### Microbial Community Analysis

2-mL samples from triplicate shake-flask enrichment cultures were centrifuged (13,000 × *g*, Microfuge, ThermoFisher Scientific, Waltham, NC, United States) and cell pellets were stored −80°C until analysis. Genomic DNA was extracted using the UltraClean DNA isolation kit (Qiagen Inc., CA, United States), following the manufacturer’s instructions. 16S-rRNA gene sequences in the enrichment cultures were analyzed by amplicon sequencing on an Illumina HiSeq sequencer (Novogene Bioinformatics Technology Co., Ltd., Beijing, China). Primers 341F (5′-CCTAYGGGRBGCASCAG-3′) and 805R (5′-GGACTACNNGGGTATCTAAT-3′) were used to generate 250 bp paired end reads and sequences were analyzed as described previously (van den Berg et al., 2017). Representative sequences for the dominant operational taxonomic unites (OTUs) (>1%) were submitted for taxonomic analysis in the SILVA database (SINA, version 1.2.11 (Pruesse et al., 2012) using default settings. The amplicon sequences of the 16S rRNA gene analysis are shown in Supplementary Table S3.

### Whole-Genome Sequencing

A 250-mL sample from a steady-state chemostat culture of *L. suebicus* LCV1 was centrifuged for 10 min at maximum speed (4700 × *g* at 4°C, Sorvall Legend XTR ThermoFisher Scientific, Waltham, NC, United States). The cell pellet was washed with TE buffer (pH 8) and stored at -20°C. DNA was extracted with the Genomic-tip 100/G Kit (Qiagen Inc., CA, United States) according to the manufacturer’s protocol except for the addition of 2.6 mg mL^–1^ zymolyase (20T, Amsbio, United Kingdom) and 4 mg mL^–1^ lysozyme (Qiagen, Hilden, Germany) to facilitate cell lysis. The amount of extracted DNA was quantified with a Qubit dsDNA BR assay kit (Thermo Fisher Scientific, Waltham, NC, United States) and its quality was assessed with NanoDrop^TM^ 2000 (Thermo Fisher Scientific, Waltham, NC, United States) and Tapestation 2200 technology (Agilent Technologies, Santa Clara, CA, United States). Sequencing was performed in-house on an Illumina MiSeq Sequencer (Illumina, San Diego, CA, United States), with MiSeq^®^ Reagent Kit v3 with 2 × 300 bp read length to obtain a 300 cycle paired-end library with an insert-size of 550 bp using TruSeq PCR-free library preparation yielding 5.92 million reads with a total quantity of 1.78 gigabase sequence. Long-read sequencing was performed with the MinION platform (Oxford Nanopore Technologies, Oxford, United Kingdom). The MinION genomic library was prepared using Nanopore 1-D ligation sequencing kit (SQK-LSK108), using 2–3 μg of input genomic DNA fragmented in a Covaris g-Tube (Covaris, Brighton, United Kingdom) with the 8–10 kb fragments settings according to manufacturer’s instructions. All R9 flow cells (FLO MIN106) were primed with priming buffer and libraries were loaded following manufacturer’s instructions. MinKNOW software (Oxford Nanopore) was used for quality control of active pores and for sequencing. Raw files generated by MinKNOW were base called using Albacore (version 1.2.5; Oxford Nanopore). Reads, in fastq format, with a minimum length of 1000 bp were extracted, yielding 1.63 Gb sequence with an average read length of 6.28 kbp.

### *De novo* Assembly

Base-calling was performed with Albacore v1.2.6 (Oxford Nanopore Technologies, Oxford, United Kingdom); FASTQ files were obtained and filtered on size (>1000 bp). The long-read genome sequences generated with the MinION platform were *de novo* assembled using Canu v1.4 (settings: genomesize = 3 m) (Koren et al., 2017); 2.78 Mbp genome into three contigs of which the largest with a length of 2.67 Mb while the two smaller with a length of 89 and 28 kbp paired-end Illumina library was aligned, using BWA (Li and Durbin, 2010), to the assembly and the resulting BAM file (Binary alignment map file) was processed by Pilon (Walker et al., 2014) for polishing the assembly (for correcting assembly errors), using correction of only SNPs and short indels (–fix bases parameter).

### Genome Annotation and Analysis

The assembled genome was uploaded to the automated Microscope platform (Vallenet et al., 2006, 2017). Manual assessment of pathway annotations was assisted by the MicroCyc (Caspi et al., 2008), Kyoto Encyclopedia of Genes and Genomes (KEGG; Kanehisa et al., 2014) and SwissProt alignment (BLASTP version 2.2.28 +; Altschul et al., 1997) databases. The genome sequence of *L. suebicus* LCV1 has been deposited at the NCBI archive with the corresponding BioProject ID PRJNA578870. Common and unique coding sequences in the two strains, with *L. suebicus* DSM5007 annotated with Prokka (Seemann, 2014), were identified with OrthoVenn2 (Xu et al., 2019).

### Proteome Analysis

A published protocol (Hansen et al., 2014) was modified to prepare whole protein extracts. Approximately 20 mg biomass (wet weight) were collected and solubilized in a solution consisting of 175 μL B-PER reagent (Illumina, San Diego, CA, United States) and 175 μL triethylammonium bicarbonate (TEAB) buffer [50 mM TEAB, 1% (w/w) sodium deoxycholate (NaDOC), adjusted to pH 8.0]. After addition of 0.1 g glass beads (acid washed, 0.1 mm diameter), cells were disrupted by bead beating with a Fast Prep FP120 (MP Biomedicals, Fisher Scientific, Hampton, NH, United States; 4 bursts of 20 s at a setting of 6 m s^–1^). Cell debris was removed by centrifugation (15 min at 14,000 × *g*) and at 4°C in a 5424R centrifuge (Eppendorf AG, Hamburg, Germany). The supernatant was transferred to a new Eppendorf tube and kept at 4°C until further processing. Proteins were precipitated by adding four volumes of ice-cold acetone. The solution was incubated at −20°C for 30 min and centrifuged 15 min at 14,000 × *g* and at 4°C in a 5424R centrifuge (Eppendorf AG, Hamburg, Germany). The protein pellet was washed twice with 200 μL ice-cold acetone and re-dissolved in 200 mM ammonium bicarbonate containing 8 M urea, to a final concentration of approximately 100 μg protein μL^–1^; 30 μL of a 10 mM dithiothreitol solution was added to 100 μL of the resulting protein solution. After 1 h incubation at 37°C, 30 μL of a freshly prepared 20 mM 3-idoleacetic acid (IAA) solution was added, followed by 30 min incubation in the dark at room temperature. The solution was then diluted to below 1 M urea with a 200 mM bicarbonate buffer. An aliquot of approximately 25 μg protein was digested overnight at 37°C using sequencing grade trypsin (Promega, Madison, WI, United States), at a trypsin-to-protein ratio of approximately 1: 50. Peptides were desalted using an Oasis HLB 96 well plate (Waters, Mildford, CT, United States) according to the manufacturer’s protocol. The purified peptide eluate was dried with a speed vac concentrator (Thermo Fisher Scientific, Waltham, NC, United States). The dried peptide fraction was resuspended in 3% acetonitrile and 0.1% formic acid solution by careful vortexing. An aliquot corresponding to approximately 50–100 ng protein digest was analyzed using a one-dimensional shot-gun proteomics [EASY nano LC connected with a QE plus Orbitrap mass spectrometer (Thermo Fisher Scientific, Waltham, NC, United States)] (Köcher et al., 2012). Raw mass spectrometry data were analyzed using PEAKS Studio X (Bioinformatics Solutions Inc.^1^) by searching against a global *L. suebicus* database downloaded from UniProtKB (July 2018). The search included a GPM crap contaminant database^2^ and used a decoy fusion for determining false discovery rates (FDRs). Search parameters included 20 ppm parent ion and 0.02 Da fragment ion mass error tolerance, up to three missed cleavage sites, carbamidomethylation as fixed and methionine oxidation and N/Q deamidation as variable modifications. Peptide spectrum matches were filtered against 1% FDR and protein identifications were accepted as being significant when at least two unique peptides were identified. The mass spectrometry proteomics data have been deposited to the Proteome Xchange Consortium via the PRIDE (Vizcaíno et al., 2016) partner repository with the dataset identifier PXD015964.

### Discontinuous Enzyme Assay

Cell extract of galacturonate-grown *L. suebicus* LCV1 (5.1 ± 0.4 g L^–1^) was incubated for 30 or 180 min at 30°C in a reaction mixture containing triethanolamine (TEA) buffer (100 mM, pH 7.6), 2.5 mM MgCl_2_.6H_2_O, 5 mM ATP, and 5 mM mannonate or no ATP and with 5 mM 6-phosphogluconate, unless stated otherwise. Samples were centrifuged (13,000 × *g*, Microfuge, ThermoFisher Scientific, Waltham, NC, United States) after incubation and the supernatant was collected; 100 μL of the supernatant was frozen at −80°C (U101 Innova freezer, Eppendorf, Hamburg, Germany) and freeze-dried over-night (Mini Lyotrap freeze-dryer, LTE Scientific Ltd., Greenfield, Oldham, United Kingdom). The supernatant was derivatized according to Niedenführ et al. (2016) without addition of AAL-mix and analyzed using a 7890A gas chromatography system (Agilent, Santa Clara, CA, United States) coupled to a 5975C MSD single quadrupole mass spectrometer (Agilent, Santa Clara, CA, United States) according to de Jonge et al. (2011); split ratio of 1: 50 for standards, split ratio of 1: 10 for samples. Identification of the peaks was done via MassHunter Workstation Qualitative Analysis software (Agilent, version B06.00) and comparison to the NIST Standard Reference Database (version 2.0).

### Enzyme-Activity Assays in Cell Extracts

Cells were harvested from steady-state chemostat cultures by centrifugation (5 min at 4696 × *g* and at 4°C, Sorvall Legend X1R, ThermoFisher Scientific, United States) and the pellet was stored at −20°C. Cells were washed and suspended in 100 mM potassium-phosphate buffer (pH 7.5) with 2 mM MgCl_2_ and protease inhibitors (complete^TM^ Protease inhibitor cocktail, Merck group, Darmstad, Germany). For LDH activity assays, 1 mM dithiothreitol was added instead of protease inhibitors. After cell disruption with a Fast Prep FP120 (MP Biomedicals, Fisher Scientific, Hampton, NH, United States; 4 bursts of 20 s at a setting of 6 m s^–1^), cell debris was removed by centrifugation [20 min at 4°C and at 20,000 r/min (rotor SS-34) Sorval RC 5C plus] and the resulting cell extract was directly used for enzyme assays. Protein concentrations were determined with the Lowry assay (Lowry et al., 1951).

Activity of LDH was measured according to van Maris et al. (2004), fructuronate reductase (UxuB) according to Linster and van Schaftingen (2004) and PDH according to Flikweert et al. (1997) in an anaerobic environment (Vinyl Anaerobic Chamber; gas phase 2.3% H_2_ and 97.7% N_2_; Coy Lab, United States) with an AvaSpec-3648 high-resolution spectrometer with AvaLight-DH-S light source (Avantes, Apeldorn, Netherlands). Aerobic enzyme-activity assays were measured at 340 nm and 30°C with a Hitachi model U-3010 spectrophotometer (Sysmex Europe GmbH, Norderstedt, Germany). Fructuronate reductase (UxuB) was measured in a reaction mixture containing TEA buffer (100 mM, pH 7.6), 2.5 mM MgCl_2_.6H_2_O, 0.6 mM NADPH, and cell extract of galacturonate-grown *L. suebicus* LCV1 (5.1 ± 0.4 g protein L^–1^). The reaction was stated with the addition of tagaturonate (0.5 mM) (Huisjes et al., 2012). 6-Phosphogluconate dehydrogenase (GndA) was measured as described previously (Scott and Abramsky, 1973), but with a 50 mM potassium-phosphate buffer (pH 7.5). The reaction was started by addition of 6-phosphogluconate (5 mM). Gluconate kinase was measured via a coupled reaction with 6-phoshogluconate dehydrogenase (0.8 U, *Saccharomyces cerevisiae*, Sigma–Aldrich, St. Louis, MO, United States). The reaction mixture contained TEA buffer (100 mM, pH 7.6), 2.5 mM MgCl_2_.6H_2_O, 1 mM ATP, 0.15 mM NADP^+^ and the reaction was started by addition of gluconate (0.5 mM, unless stated otherwise). The same assay was used for estimation of mannonate-kinase activities, by starting the reaction with mannonate (0.2 mM unless stated otherwise) instead of gluconate and using native *L. suebicus* 6-mannonate 2-epimerase activity in cell extracts as coupling enzyme. All assays were performed in duplicate and reaction rates were proportional to the amount of cell extract added.

## Data Availability Statement

The datasets generated for this study can be found in the NCBI archive BioProject ID PRJNA578870, PRIDE repository PXD015964.

## Author Contributions

ML, JP, and LV designed the experiments, interpreted the results, and wrote the manuscript. LV performed the enrichment, isolation, and bioreactor experiments. LV and MB analyzed the *L. suebicus* genome. ML and LV performed the continuous and discontinuous enzymatic assays. MP and CR performed and interpreted MS studies on proteome and metabolites, respectively. All authors read and approved the final manuscript.

## Conflict of Interest

The authors declare that the research was conducted in the absence of any commercial or financial relationships that could be construed as a potential conflict of interest.

## Footnotes

1 http://www.bioinfor.com

2 https://www.thegpm.org/crap/
