# Supplementary material for: A Novel D-Galacturonate Fermentation Pathway in Lactobacillus suebicus Links Initial Reactions of the Galacturonate-Isomerase Route With the Phosphoketolase Pathway
Source: Front Microbiol. 2020 Jan 17;10:3027. doi: 10.3389/fmicb.2019.03027 (PMC6978723; doi:10.3389/fmicb.2019.03027)
Supplement: Supplementary file 1 [file Table_1.docx]

# Supplemental material

Figure S1 | Absolute counts of 16S-rRNA gene amplicon analysis of three independent anaerobic enrichments on d-galacturonate (pH 4), supplemented with 0.1 g L^-1^ yeast extract. Enrichment cultures were inoculated with a mixture of rotting orange peels, cow-rumen content and orange-peel-enriched compost. Red bars represent *Lactobacillus* genus, green bar represents the *Clostridium* sensu stricto 12 genus and blue bars represent the combined operational taxonomic units (OTUs) with an abundance below 1 % (others).

Table S1 | Anaerobic growth and catabolic products of *Lactobacillus suebicus* LCV1 on different carbon sources in synthetic media with 4 g L^-1^ carbon source, supplemented with 0.4 g L^-1^ yeast extract. All experiments were performed in an anaerobic chamber (gas phase 5 % CO_2_, 5 % H_2_, and 90 % N_2_) at 25 ^o^C in shake flasks at pH 4. Growth was measured as optical density at 660 nm upon carbon depletion, along with concentrations of catabolic products. Shake flasks were incubated for 12 d. Standard errors were derived from duplicate experiments. N.d.: ‘not detected’.

|  | **Optical Density**  **at 660nm** | **Lactate (mM)** | **Acetate (mM)** | **Ethanol (mM)** |
| --- | --- | --- | --- | --- |
| Galacturonate | 0.68 ± 0.01 | 18.0 ± 0.1 | 19.5 ± 0.0 | 3.8 ± 0.7 |
| Glucose | n.d. | n.d. | n.d. | n.d. |
| Arabinose | 0.94 ± 0.00 | 23.2 ± 0.1 | 23.1 ± 0.0 | 6.2 ± 0.1 |
| Gluconate | 0.80 ± 0.07 | 17.3 ± 0.3 | 7.8 ± 0.2 | 22.5 ± 0.2 |
| Glucuronate | 0.80 ± 0.02 | 18.6 ± 0.0 | 20.2 ± 0.1 | 7.0 ± 0.2 |
| Pyruvate | n.d. | n.d. | n.d. | n.d. |

**Table S2** | Statistical data for the assembled and annotated genome sequence of *Lactobacillus suebicus* LCV1 and *Lactobacillus suebicus* DSM 5007 (Nam et al., 2011).

|  | ***Lactobacillus suebicus* LCV1** | ***Lactobacillus suebicus DSM 5007*** |
| --- | --- | --- |
| **Genome size (Mbp)** | 2.8 | 2.6 |
| **Scaffolds** | 3 | 143 |
| **Contigs** | 3 | 143 |
| **Contigs N50 (bp)** | 2673450 | 54666 |
| **Max contig size (bp)** | 2673450 | 151146 |
| **GC content (%)** | 39.1 | 39.0 |
| **Protein coding density (%)** | 85.5 | n.d. |
| **Coding sequences (CDS)** | 2811 | 2543 |
| **Unique CDS** | 149 | 8 |
| **rRNA copies** | 6 | 3 |


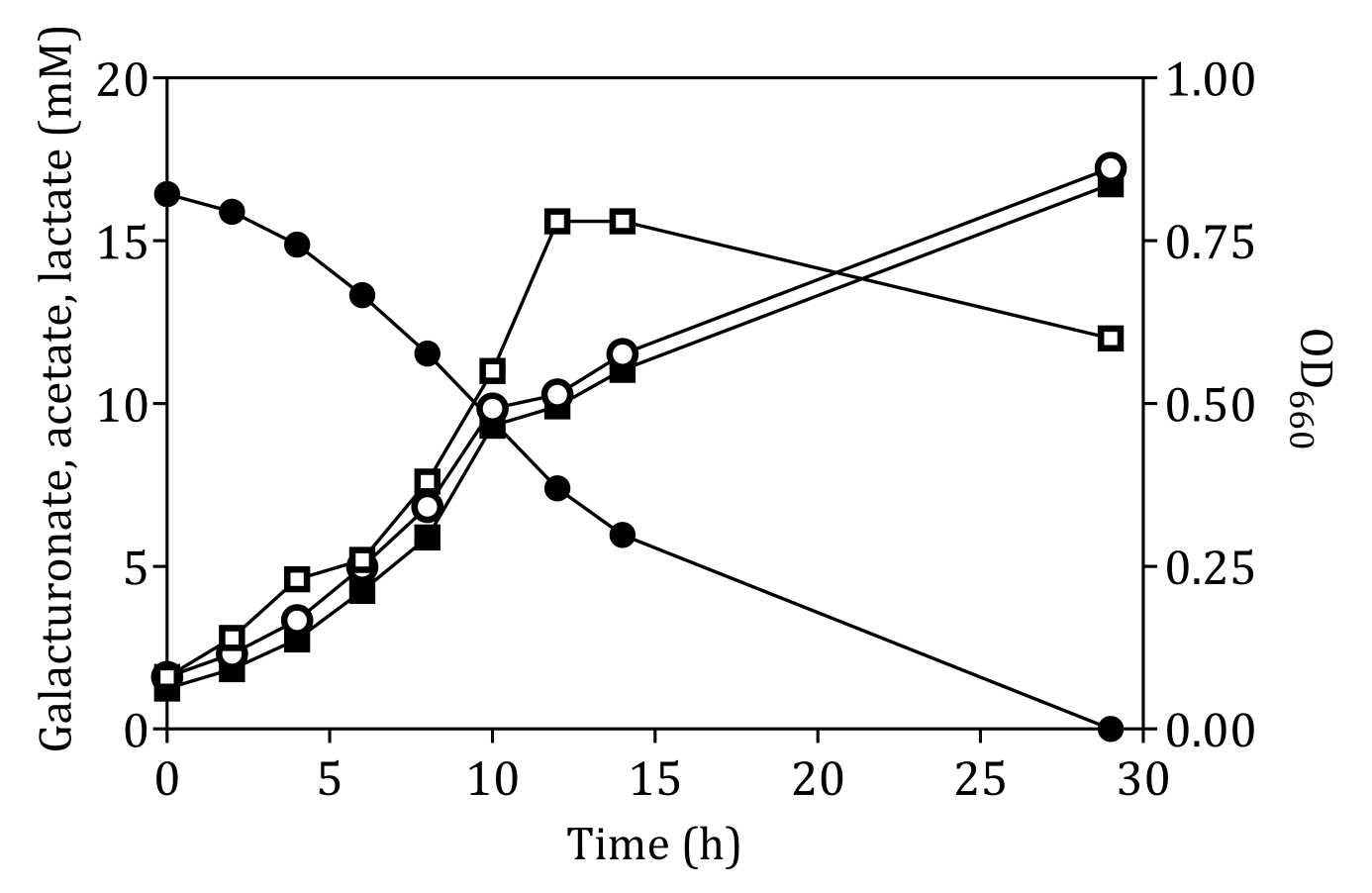


Figure S2 | Anaerobic growth and product formation of an anaerobic bioreactor batch culture of *L. suebicus* LCV1 on d-galacturonate (3.3 g L^-1^) at pH 4 and at 30 ^o^C. Symbols: ⚫ d-galacturonate, ◼ acetate, 🞏 lactate and ⭘ optical density. The data shows one of two independent replicates, bioreactor 2.


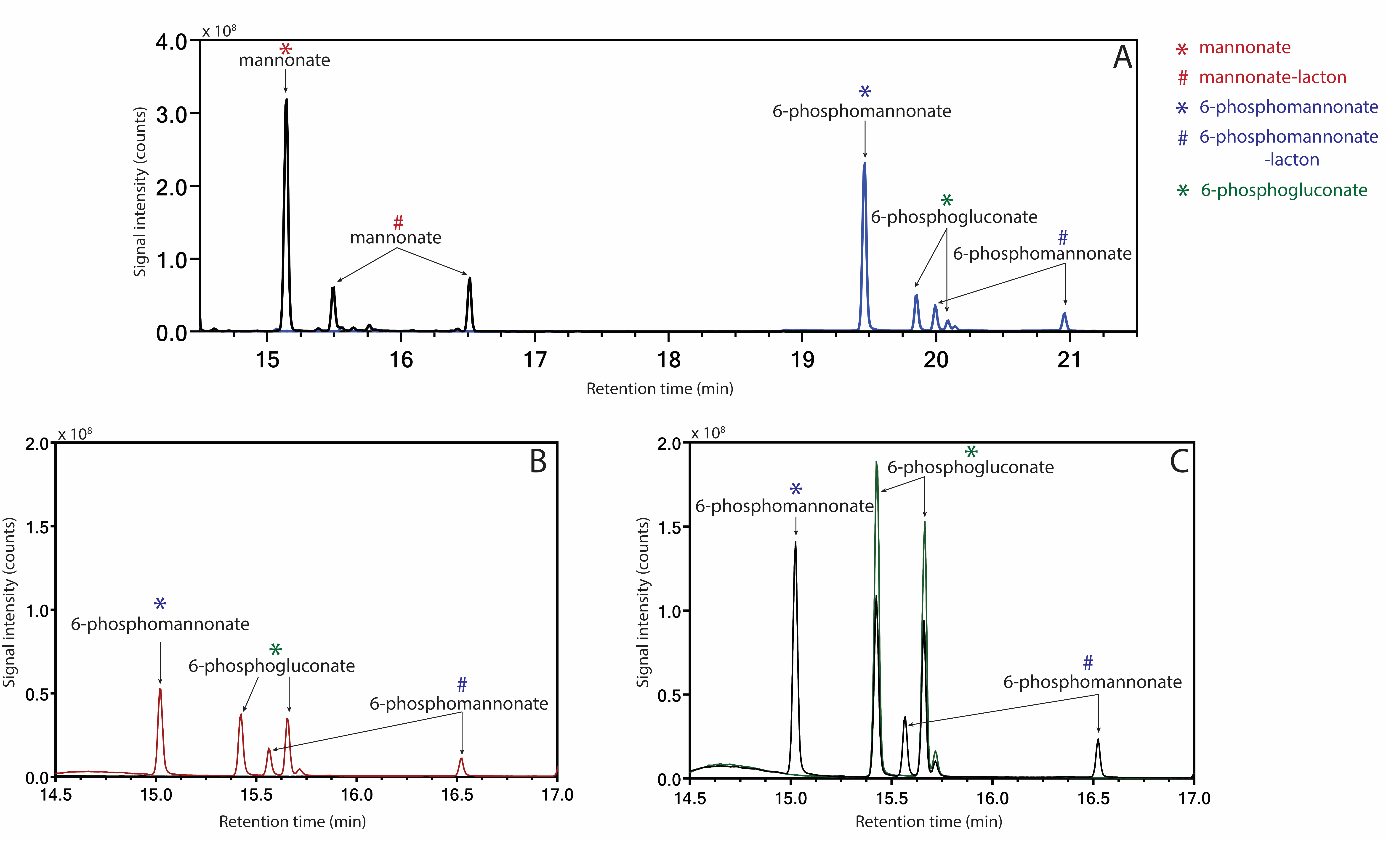


Figure S3 | Identification of products from the conversion of mannonate or 6-phosphogluconate in the presence and absence of ATP with cell extract from galacturonate-grown *L. suebicus* LCV1 (D=0.13 h^-1^, T= 30 ^o^C and pH 4). Panel A) 0.5 h incubation, mannonate with cell extract and no ATP (black line) and ATP (blue line), B) mannonate with cell free extract and ATP, start of the incubation (black line) and 3h incubation (red line), C) 6-phosphogluconate with cell extract start of the incubation (green line) and 3h incubation (black line). One of two of independent biological duplicate experiments are shown. The retention times of the compounds differ between panel A and B, C due to shortening of the column for maintenance purposes.


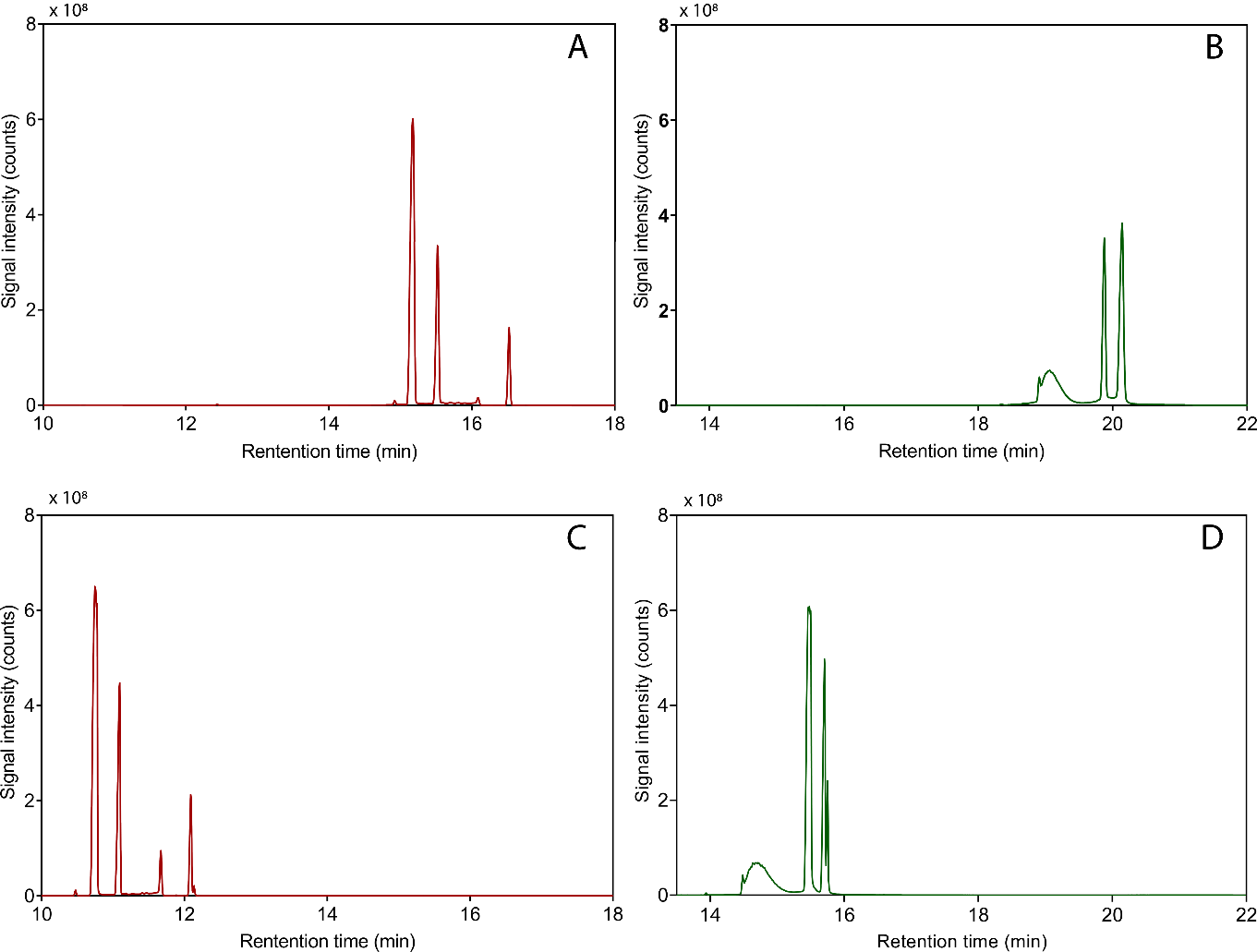


Figure S4 | Signal intensities in counts and retention times in minutes of the standards used for the GC-MS analysis before and after reduction of the column. A) mannonate standard before reduction. B) 6-phosphogluconate standard before reduction. C) mannonate standard after reduction. D) 6-phosphogluconate standard after reduction.

**Peak 1**

**Peak 2**

**Peak 3***

**Peak 4**

**Retention time (min)**

**Signal intensity (counts)**

**Peak 1**

**Peak 2**

**Peak 4**

**Mannonate (syn-form)**

**Lactone form (proposed D)**

*** Abundance too low for NIST match,**

**proposed mannonate (anti-form)**

**Lactone form (proposed L)**

**Signal intensity (counts)**

**Mannonate standard (oximated/silylated)**

**m/z**

Figure S5 | The annotated GC-MS profile of mannonate with the signal intensity in counts and the mass spectrometry profiles for each peak in m/z.


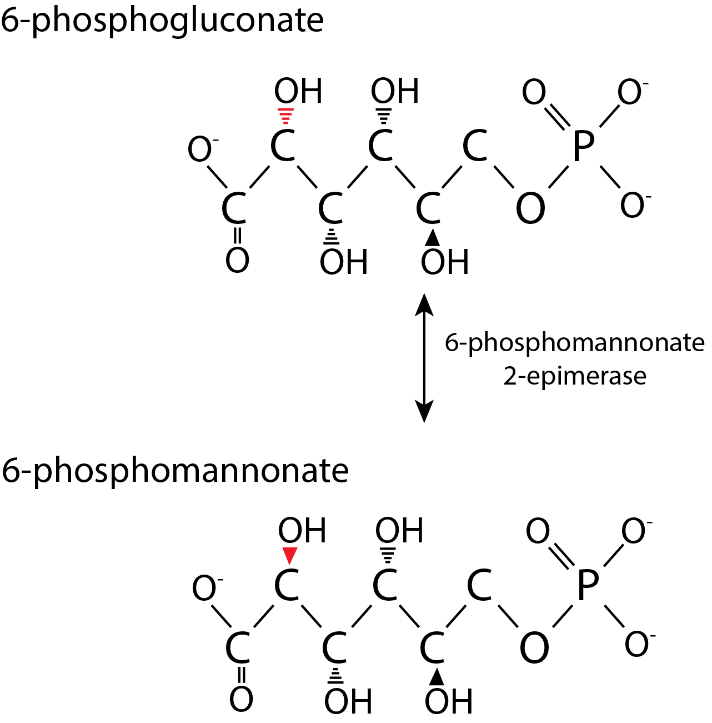


Figure S6 | The proposed reaction catalysed by 6-phosphomannonate 2-epimerase, with the affected hydroxyl moiety depicted in red.

Table S3 | 16S rRNA gene amplicon sequences of the genera shown in Figure S1

| **Genus** | **Amplicon sequence** |
| --- | --- |
| *Lactobacillus* | TAGGGAATCTTCCACAATGGGCGCAAGCCTGATGGAGCAACACCGCGTGAGTGAAGAAGGGTTTCGGCTCGTAAAACTCTGTTGTTAAAGAAGAACGTATCTAAGAGTAACTGCTTAGGTAGTGACGGTATTTAACCAGAAAGCCACGGCTAACTACGTGCCAGCAGCCGCGGTAATACGTAGGTGGCAAGCGTTATCCGGATTTATTGGGCGTAAAGCGAGCGCAGGCGGTTttttAAGTCTGATGTGAAAGCCTTCGGCTTAACCGAAGAAGTGCATCGGAAACTGGGAGACTTGAGTGCAGAAGAGGACAGTGGAACTCCATGTGTAGCGGTGAAATGCGTAGATatatGGAAGAACACCAGTGGCGAAGGCGGCTGTCTGGTCTGTAACTGACGCTGAGGCTCGAAAGCATGGGTAGCGAACAGG |
| *Clostridium* sensu stricto 12 | TGGGGAATATTGCACAATGGGCGAAAGCCTGATGCAGCAACGCCGCGTGAGTGATGACGGTCTTCGGATTGTAAAGCTCTGTCTTTTGGGACGATAATGACGGTACCAAAGGAGGAAGCCACGGCTAACTACGTGCCAGCAGCCGCGGTAATACGTAGGTGGCAAGCGTTGTCCGGATTTACTGGGCGTAAAGGATGTGTAGGCGGATACTTAAGTGAGATGTGAAAGCCCCGAGCTTAACTTGGGGACTGCATTTCAAACTGGGTGTCTAGAGTGCAGGAGAGGAAAGCGGAATTCCTAGTGTAGCGGTGAAATGCGTAGAGATTAGGAAGAACATCAGTGGCGAAGGCGGCTTTCTGGACTGTAACTGACGCTGAGGCATGAAAGCGTGGGGAGCAAACAGG |
